# Detection of Serum Protein Biomarkers for the Diagnosis and Staging of Hepatoblastoma

**DOI:** 10.3390/ijms160612669

**Published:** 2015-06-04

**Authors:** Wei Zhao, Juan Li, Junjie Zhang, Pengfei Gao, Hang Pei, Lei Wang, Fei Guo, Jiekai Yu, Shu Zheng, Jiaxiang Wang

**Affiliations:** 1 Department of Pediatric Surgery, the First Affiliated Hospital of Zhengzhou University, Jianshe Road, Zhengzhou 450000, China; E-Mails: 13460300188@163.com (W.Z.); zhangjunjiezzu@126.com (J.Z.); gaopengfeizzu@126.com (P.G.); peihangzzu@163.com (H.P.); wangleizzuff@126.com (L.W.); guofeizzu@126.com (F.G.); 2 Department of Respiratory Medicine, the First Affiliated Hospital of Zhengzhou University, Jianshe Road, Zhengzhou 450000, China; E-Mail: lijuan19881027@163.com; 3 Institute of Cancer, the Second Affiliated Hospital, College of Medicine, Zhejiang University, Jiefang Road, Hangzhou 310000, China; E-Mails: yujiekaizju@163.com (J.Y.); zhengshuzju@163.com (S.Z.)

**Keywords:** hepatoblastom, SELDI-TOF-MS, MALDI-TOF-MS, 2D-LC-LTQ-MS, apolipoprotein A–I, protein biomarker

## Abstract

The present study aimed to identify serum biomarkers for the detection of hepatoblastoma (HB). Serum samples were collected from 71 HB patients (stage I, *n* = 19; stage II, *n* = 19, stage III, *n* = 19; and stage IV, *n* = 14) and 23 age- and sex-matched healthy children. Differential expression of serum protein markers were screened using surface-enhanced laser desorption/ionization time-of-flight mass spectrometry (SELDI-TOF-MS), and the target proteins were isolated and purified using HPLC and identified using matrix-assisted laser desorption/ionization time-of-flight mass spectrometry (MALDI-TOF-MS), SEQUEST, and bioinformatics analysis. Differential protein expression was confirmed by enzyme-linked immunosorbent analysis (ELISA). SELDI-TOF-MS screening identified a differentially expressed protein with an *m*/*z* of 9348 Da, which was subsequently identified as Apo A–I; its expression was significantly lower in the HB group as compared to the normal control group (1546.67 ± 757.81 *vs.* 3359.21 ± 999.36, respectively; *p* < 0.01). Although the expression level decreased with increasing disease stage, pair-wise comparison revealed significant differences in Apo A–I expression between the normal group and the HB subgroups (*p* < 0.01). ELISA verified the reduced expression of Apo A–I in the HB group. Taken together, these results suggest that Apo A–I may represent a serum protein biomarker of HB. Further studies will assess the value of using Apo A–I expression for HB diagnosis and staging.

## 1. Introduction

Hepatoblastoma (HB) is the most common form of liver cancer in children, accounting for approximately 80% of liver cancers and 1% of malignant tumors in children [1,2]. HB is the third most common childhood abdominal malignancy following neuroblastoma and Wilms’ tumor [3], and >90% of hepatoblastoma occur under the age of four years and there is a male predominance [4]. Among children under the age of five years, HB accounts for approximately 91% of all liver malignancies, affecting 0.5–1.5 per million children per year [5].

HB is composed of malignant tumor cells derived from differentiated and proliferating pluripotent stem cells during embryonic development [6,7]. Early diagnosis and treatment can significantly improve the survival rate of children with HB [8]; however, HB does not induce specific symptoms. Most children are admitted to the hospital due to a large abdominal mass and already have stage II or greater disease; some patients even have metastases to the hilus hepatis, portal vein, and brain at diagnosis [9]. At present, auxiliary examinations of HB mainly include color Doppler ultrasound, computed tomography (CT) and hepatic arteriography; however, none of these methods can diagnose early HB. Therefore, identification of a method for the early diagnosis of HB is crucial for impacting the prognoses of HB patients.

Surface-enhanced laser desorption/ionization time-of-flight mass spectrometry (SELDI-TOF-MS) technology, matrix-assisted laser desorption/ionization time-of-flight mass spectrometry (MALDI-TOF-MS) technology, protein chip technology, the yeast two-hybrid system and bioinformatics analysis have been widely applied to identify and analyze protein structure and function [10,11]. Proteomics has also been applied in oncology to identify tumor-related markers as a tool for the early diagnosis and treatment of specific diseases [12,13], including Wilms’ tumor, ovarian cancer, prostate cancer, pancreatic cancer, colon cancer, and breast cancer [14,15,16,17,18,19,20,21]. The present study sought to identify a more accurate serum protein biomarker for HB diagnosis and staging by screening serum samples from HB patients and healthy control children.

## 2. Results and Discussion

### 2.1. Screening of Differentially Expressed Protein Markers

**Figure 1:**
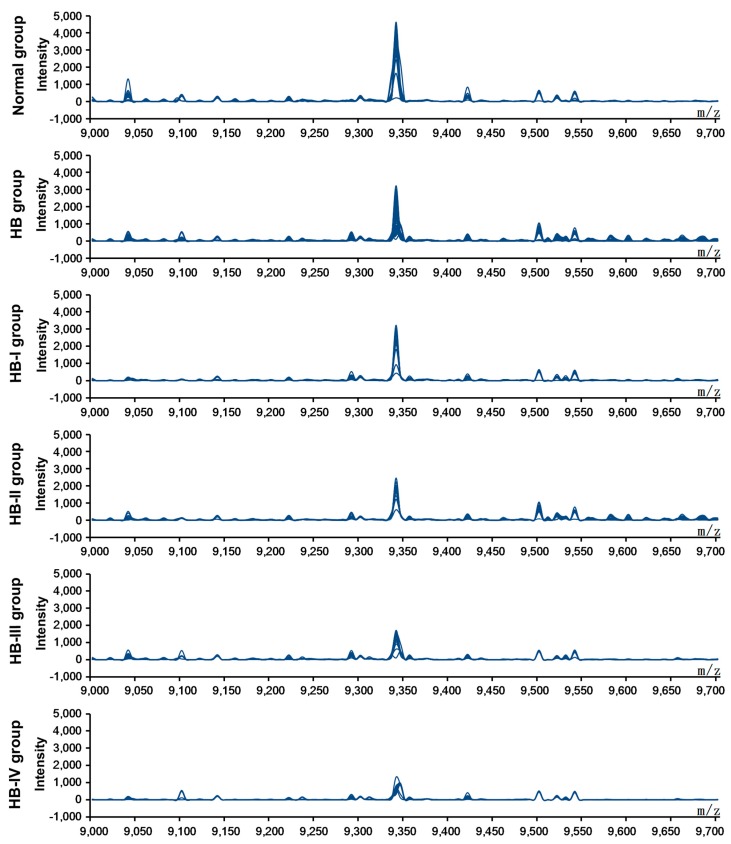
SELDI-TOF-MS analysis of proteins or peptide segments with an *m*/*z* of 9348 Da in the normal and HB groups.

**Figure 2:**
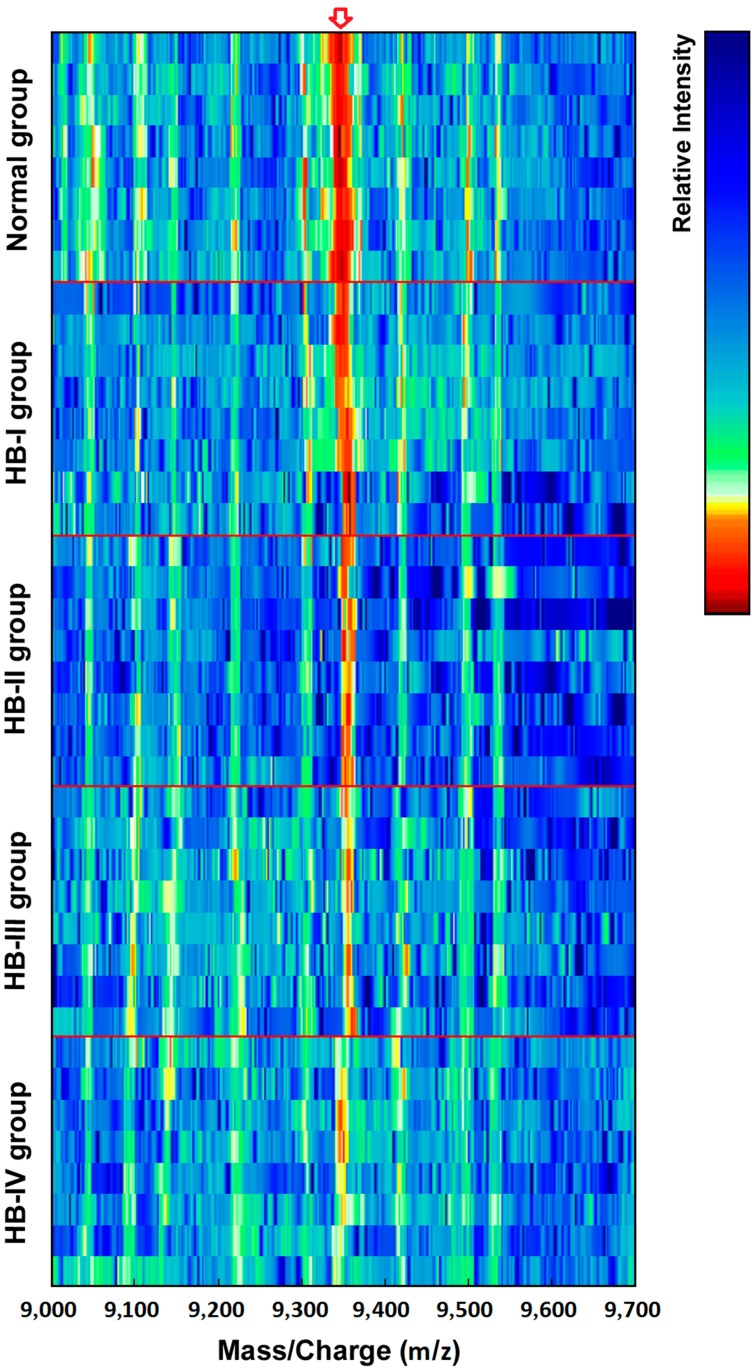
Simulated electrophoretogram of proteins or peptide segments with an *m*/*z* of 9348 Da in the normal and HB groups.

After screening the pretreated sera from the healthy children and HB patients using SELDI-TOF-MS, data from each protein peak and the peak values of the decomposed peptides were obtained. These protein peak values were standardized and analyzed using the Wilcoxon rank-sum test, and comparisons between the normal and HB groups were undertaken. Ten differentially expressed proteins, including four upregulated and six down-regulated protein peak values, were observed in the serum of HB patients (Table 1). A composite model with the highest Youden index was screened using the support vector machine (SVM) to obtain a protein marker with a Mass-to-charge ratio (*m*/*z*) of 9348 Da (Figure 1). The expression level of this marker was 3359.21 ± 999.36 and 1546.67 ± 757.81 in the normal control and HB groups, respectively (*p* < 0.01; Table 2). Analysis of the HB group by disease stage revealed that the expression level of the protein marker with an *m*/*z* of 9348 Da was significantly lower at each disease stage as compared with the normal group (*p* < 0.01; Table 2). Moreover, there were significant differences between HB subgroups (*p* < 0.01; Table 2). Figure 2 shows simulated electrophoretogram of proteins or peptide segments with an *m*/*z* of 9348 Da in the normal and HB groups with SELDI-TOF-MS. Using the method of leave-1-out for cross detection, the sensitivity of discriminating 71 HB and 23 normal subjects was 98.32%, and its specificity was 87.96%.

**Table 1:** The ten differentially expressed proteins in hepatoblastoma *vs.* normal (mean ± SD).

*m*/*z* (Da)	Hepatoblastoma	Normal
2032.3	889.41 ± 106.21	1058.41 ± 214.65
2486.6	621.45 ± 187.24	1306.64 ± 323.41
2963.5	2132.69 ± 524.12	2654.73 ± 408.64
3786.1	1135.30 ± 284.77	1462.58 ± 367.59
4571.3	1659.73 ± 325.38	974.57 ± 236.87
5269.0	1237.29 ± 363.74	1687.02 ± 397.54
5457.2	884.30 ± 204.23	656.31 ± 146.09
9348.0 *	1546.67 ± 757.81	3359.21 ± 999.36
16,589.3	871.62 ± 659.37	452.14 ± 109.43
18,946.2	2068.85 ± 574.33	1564.89 ± 337.57

* The diagnostic model with the highest Youden index comprising *m*/*z* peak of 9348 Da.

**Table 2:** SELDI-TOF-MS mass spectrometry analysis of proteins or peptide segments with an *m*/*z* of 9348 Da in the normal and HB groups.

Group	Case	Intensity	*p* Value			
			Normal	HB-I	HB-II	HB-III
Normal	23	3359.21 ± 999.36	–	–	–	–
HB	71	1546.67 ± 757.81	0.000000	–	–	–
HB-I	19	2324.12 ± 679.42	0.000432	–	–	–
HB-II	19	1718.90 ± 418.62	0.000000	0.002153	–	–
HB-III	19	1268.10 ± 399.09	0.000000	0.000001	0.001674	–
HB-IV	14	635.91 ± 237.56	0.000000	0.000000	0.000000	0.000010

### 2.2. Purification and Identification of the Target Proteins

#### 2.2.1. Purification of the Target Proteins

**Figure 3:**
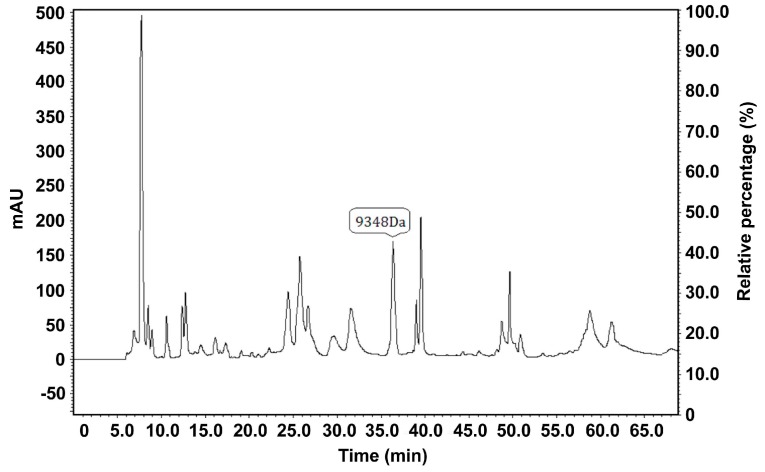
Isolation and purification of the proteins or peptide segments with an *m*/*z* of 9348 Da by HPLC. MALDI-TOF-MS confirmed that the sample eluted at minutes 36 and 37 contained the proteins with an *m*/*z* of 9348 Da.

**Figure 4:**
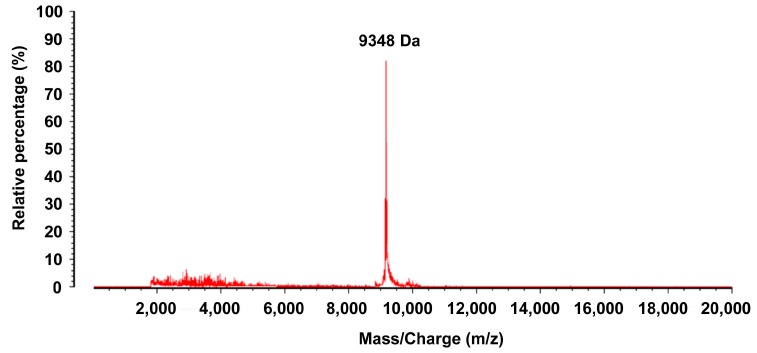
Isolation and purification of the proteins or peptide segments with an *m*/*z* of 9348 Da by HPLC. MALDI-TOF-MS confirmed that the sample eluted at minutes 36 and 37 contained the proteins with an *m*/*z* of 9348 Da.

Serum samples with relatively high levels of the target protein expression were used for subsequent isolation and purification. Each protein having a peak value as detected by high performance liquid chromatography (HPLC) was collected (Figure 3) and subsequently analyzed by MALDI-TOF-MS (Figure 4). Regarding the protein with an *m*/*z* of 9348 Da, the difference between the MALDI-TOF-MS and SELDI-TOF-MS analyses was 0.3%.

#### 2.2.2. Identification of the Target Proteins

**Figure 5:**
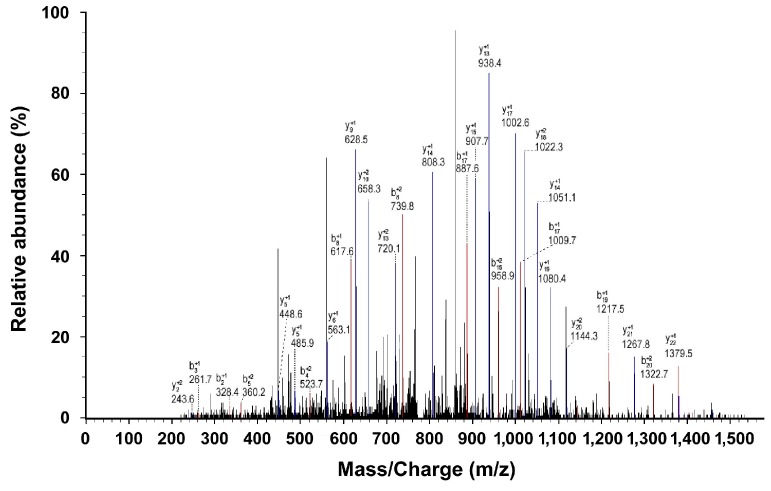
2D-LC-LTQ-MS analysis of peptide segments obtained by enzymatic hydrolysis of the proteins or peptide segments with an *m*/*z* of 9348 Da.

The protein sample with an *m*/*z* of 9348 Da was digested, and the Peptide mass fingerprints (PMFs) of the target protein was obtained using two-dimensional liquid-chromatography linear-trap-quadrupole mass-spectrometry (2D-LC-LTQ-MS) (Figure 5). After the amino acid sequences of the various protein fragments were obtained (Table 3), they were recombined to obtain a complete amino acid sequence (Table 4). Analysis of the sequence using the SEQUEST program and the Bioworks database identified Apolipoprotein A–I (Apo A–I) with a matching rate of 45.0% and a matching score of 88 points.

**Table 3:** Amino acid sequence of each peptide yielded by protein digestion as determined by 2D-LC-LTQ-MS.

*m*/*z* (Da)	Protein	Peptides Identified	Sequence
9348	Apolipoprotein A–I	K.WQEEMELYRQK.V	K**WQEEMELYRQKVEPLRAELQEGARQK**LHELQEK**LSPLGEEMR**DRAR**AHVDALRTHLAPYSDELR**QR**LAARLEALK**ENG
		R.QKVEPLR.A	
		R.AELQEGARQK.L	
		K.LSPLGEEMR.D	
		R.AHVDALR.T	
		R.THLAPYSDELR.Q	
		R.LAARLEALK.E	

Peptides identified: The target protein was dissociated by enzymatic hydrolysis into little peptides, and these little peptides were identified by 2D-LC-LTQ-MS; The amino acid sequences of these little peptides were matched with the amino acid sequence of Apo C-I, and the equal parts were showed in boldface.

**Table 4:** Amino acid sequence of the full-length protein obtained by matching and recombination of peptides.

*m*/*z* (Da)	Protein	Confirmed Peptide	Coverage Rate	Score
9348	Apolipoprotein A–I	WQEEMELYRQKVEPLRAELQEGARQKLHELQEKLSPLGEEMRDRARAHVDALRTHLAPYSDELRQRLAARLEALK	45.0%	88

### 2.3. Verification of Apo A–I Expression Using Enzyme-Linked Immunosorbent Analysis (ELISA)

**Figure 6:**
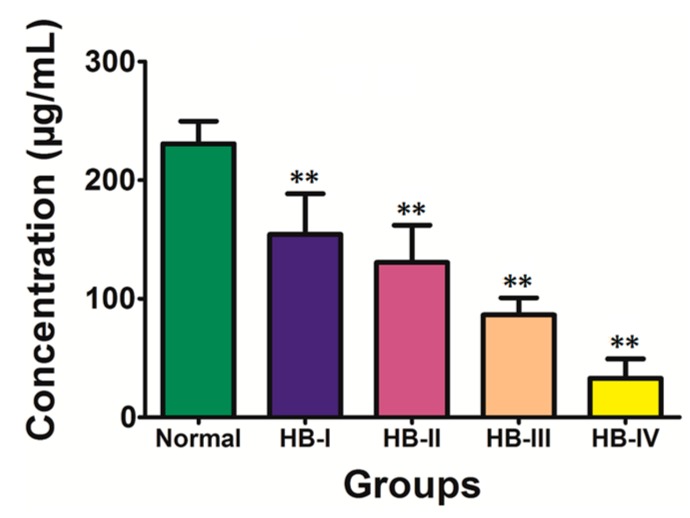
Serum Apo A–I protein levels in the normal healthy children and HB patients. Apo A–I levels were determined by ELISA. ** represents a significantly different change relative to the normal group, *p* < 0.01.

To verify that the target protein identified by MALDI-TOF-TOF was Apo A–I, we analyzed the Apo A–I protein expression in the sera of the normal control and HB groups by ELISA. As shown in Figure 6, the concentration of Apo A–I in the normal group was significantly higher than all of the HB subgroups (230.65 ± 18.92 *vs.* 154.14 ± 34.45, 130.51 ± 31.37, 86.32 ± 14.44 and 32.87 ± 16.44 μg/mL, respectively; *p* < 0.01; Figure 6).

### 2.4. Specificity and Sensitivity of the Biomarker

Serum samples of 32 HB patients and 29 healthy children were gathered by blinding method, all of which were diagnosed by pathology. To research the sensitivity and the specificity, the protein biomarker APO C–I, α-fetoprotein and computed tomography were used to diagnose the disease of these samples. The results of various kinds of diagnostic methods are shown in Table 5.

**Table 5:** Sensitivity and specificity of various diagnosis methods between HB and healthy children.

Diagnosis	Number of Cases		Sensitivity	Specificity
	HB Group	Healthy Group		
Pathology	32	29	100% (32/32)	100% (29/29)
Apo C–I	32	29	96.88% (31/32)	95.55% (28/29)
AFP	32	29	53.13% (17/32)	86.21% (25/29)
CT	32	29	68.75% (22/32)	100% (29/29)

Serum samples of 32 HB patients and 31 patients with other pediatric abdominal solid tumors (including 14 cases of Wilms’ tumor, 10 cases of neuroblastoma and seven cases of malignant teratoma) were gathered by blinding method, all of which were diagnosed by pathology. To research the sensitivity and the specificity, the protein biomarker APO C–I, α-fetoprotein and computed tomography were used to diagnose the disease of these samples. The results of various kinds of diagnostic methods are shown in Table 6.

**Table 6:** Sensitivity and specificity of various diagnosis methods between HB and other pediatric abdominal solid tumors.

Diagnosis	Number of Cases		Sensitivity	Specificity
	HB Group	Other Tumors		
Pathology	32	31	100% (32/32)	100% (31/31)
Apo C–I	32	31	96.88% (31/32)	87.10% (27/31)
AFP	32	31	53.13% (17/32)	61.29% (19/31)
CT	32	31	68.75% (22/32)	100% (31/31)

### 2.5. Discussion

In the current study, we used SELDI-TOF-MS to identify a HB serum protein biomarker with a peak *m*/*z* value of 9348 Da, Apo A–I, which was significantly reduced in the serum of HB patients relative to that observed in healthy children. Apo A–I expression was also significantly reduced in the serum of children with each stage of HB compared with the normal group, and its expression level was relatively low in the groups with high stages of HB. Thus, Apo A–I may be a diagnostic and prognostic biomarker of HB.

Apo A–I, a subtype of Apo A and the most important structural protein of high density lipoprotein (HDL), accounts for 80%–90% of HDL and is the main component of the Apo A family. Apo A–I takes part in many steps of lipid metabolism and affects the body’s metabolism and development of chronic diseases. The liver participates in conversion of the pre-Apo A–I into mature Apo A–I; therefore, liver injury or liver tumors inhibit production of Apo, altering plasma lipid levels and lipoprotein structure [22]. Thus, Apo A–I is related to the severity of liver disease. Moreover, disorders of lipoprotein metabolism in the serum of patients with malignant tumors have been noted. For example, plasma Apo A–I is related to the development of many tumors, such as stomach cancer, ovarian cancer, leukemia, and colon cancer [23,24]. Furthermore, serum HDL and Apo A–I levels are significantly reduced in these patients with cancer [25], which may be due to the following: (1) Increased HDL uptake by pathological monocytes; (2) Decreased surface activity of HDL; and (3) The increased demand for cholesterol for the synthesis of cell membranes to support the high level of cell proliferation characteristic of tumorigenesis [26].

Other studies have found a relationship between Apo A–I expression with malignant tumors. It is a specific serum biomarker of early-stage ovarian cancers with diagnostic value [24]. In a nude mouse model of ovarian cancer, Apo A–I mimetic peptides increased their survival rate [27], and the short 18 amino acid Apo A–I mimetic peptide can regulate the expression of hypoxia-inducible factor (HIF-1α), inhibit the formation of the vascular endothelial growth factor (VEGF) and tumor angiogenesis, and induce apoptosis of ovarian cancer cells [28,29]. Furthermore, increased plasma Apo A–I levels could reduce the course of gastric cancer in mice and was negatively related to the size of tumor [30].

Cancer involves multiple genes and results from complex interactions between oncogenes and tumor suppressor genes. Gene mutations or abnormal regulation of gene expression may alter the expression level or structure of cellular proteins. Changes in these proteins may thus serve as markers, reflecting dynamic changes in oncogenes, tumor suppressor genes, and the interactions between them, as well as tumor load, tumor resection, recurrence, and metastasis. In our study, the expression of Apo A–I is lower in children with HB than in normal controls, suggesting that HB inhibits the expression of Apo A–I. In addition, Apo A–I expression was reduced significantly when HB staging further deteriorated, suggesting that this inhibition of Apo A–I expression was stronger when the progress of HB disease was more serious. Of course, we can only infer that HB inhibits the expression of Apo A–I from one direction. However, we need further trials to speculate whether the abnormal expression of Apo A–I led to the occurrence and development of HB.

In conclusion, Apo A–I was identified to be a serum protein marker of HB in the present study, and this marker may be an indicator of predicting HB stage. Nevertheless, the present study results are not sufficient to determine Apo A–I as the only clinical diagnostic criteria, because Apo C–I was detected between the HB group and normal group in this study without considering its involvement in other diseases. Further studies will include a larger number of samples to confirm its specificity and sensitivity and compare it with other diagnostic methods currently used in clinical practice. In addition, the relationship between Apo A–I and HB will be further explored to determine the effect of Apo A–I expression on the pathogenesis of HB.

## 3. Experimental Section

### 3.1. Equipment and Reagents

WCX2 Protein Chip, Ciphergen Biosystems Inc., Fremont, CA, USA; PBS II+ SELDI-TOF-MS, Ciphergen Biosystems Inc.; Bioprocessor, Ciphergen Biosystems Inc.; SPD SpeedVac, Thermo Electron Inc., Waltham, MA, USA; High performance liquid chromatography, Shimadzu Inc., Kyoto, Japan; Trypsin, Promega Inc., Madison, WI, USA; MALDI-TOF-TOF, Bruker Inc., Karlsruhe, Germany; 2D-LC-LTQ-MS, Thermo Electron Inc.; Human Apo A–I ELISA Kit, BOSTER, Wuhan, China; Thermo Scientific Varioskan Flash, Thermo Electron Inc.

### 3.2. Clinical Data

All serum samples were collected from patients admitted to the First Affiliated Hospital of Zhengzhou University between December 2007 and December 2013. Serum samples of 23 healthy children were included as the normal control group and included 11 males and 12 females with an average age of 61.57 ± 21.82 months (range, 28–102 months). Serum samples from 71 HB patients were included in the HB group, including 33 males and 38 females with an average age of 59.54 ± 17.05 months (range, 16–96 months). According to the PRE-Treatment tumor EXTension (PRETEXT) guidelines, 19 had stage I disease, 19 had stage II disease, 19 had stage III disease and 14 had stage IV HB (HB-I, HB-II, HB-III and HB-IV groups, respectively), and all of these were confirmed with pathological diagnosis by two pathologists. Patient characteristics were obtained from the patients’ medical records and are shown in Table 7. The serum samples from the patients with hepatoblastoma were collected prior to the start of treatment including chemotherapy and resection. All serum samples were collected between 05:00 AM to 06:00 AM in a fasting state, after which they were incubated at room temperature for 1 h and centrifuged at 3000 rpm for 20 min. The supernatant was collected and preserved at −80 °C until further use. Before the study, informed consent was obtained from the parents of these children. This study was approved by the Ethics Committee of Zhengzhou University.

**Table 7:** Amino acid sequence of the full-length protein obtained by matching and recombination of peptides.

Patient ID	Age (Months)	Sex	Stage *	Tumor Size (cm)	Metastasis	Patient ID	Age (Months)	Sex	Stage	Tumor Size (cm)	Metastasis
P24	46	F	I (R)	4.5	None	P60	41	F	II (L)	4.5	None
P25	59	F	I (R)	4	None	P61	48	M	II (L)	5	None
P26	86	F	I (R)	3	None	P62	72	M	III (L)	8.5	None
P27	76	M	I (R)	5	None	P63	36	F	III (R)	7	None
P28	84	F	I (R)	4.5	None	P64	54	F	III (R)	9	None
P29	64	M	I (R)	3	None	P65	93	F	IIIa	6 + 3.5 **	Intraperitoneal
P30	95	M	I (L)	3	None	P66	47	M	IIId	8	None
P31	70	M	I (R)	3.5	None	P67	62	M	IIId	6	None
P32	37	F	I (L)	3	None	P68	42	F	III (R)	7.5	None
P33	16	M	I (R)	4	None	P69	63	M	III (R)	8	None
P34	82	M	I (L)	2.5	None	P70	40	F	IIId	5.5	None
P35	59	M	I (L)	3	None	P71	39	M	III (L)	6	None
P36	62	M	I (L)	2.5	None	P72	74	F	IIIa	5 + 3 **	None
P37	72	F	I (R)	4	None	P73	53	M	III (L)	7	None
P38	45	F	I (L)	3	None	P74	66	F	III (R)	8.5	Intraperitoneal
P39	62	F	I(R)	4	None	P75	39	F	III (R)	6.5	None
P40	36	M	I (L)	2	None	P76	48	M	IIId	7.5	Intraperitoneal
P41	52	F	I (L)	3	None	P77	58	M	IIId	7	None
P42	49	F	I (L)	3	None	P78	46	F	IIId	7	None
P43	85	F	IIa	3.5 + 2 **	None	P79	68	M	III (L)	9	None
P44	83	M	II (L)	6	None	P80	60	F	IIId	8	None
P45	74	M	II (R)	5.5	None	P81	54	M	IV	9.5	Extraperitoneal
P46	96	F	II (R)	7	None	P82	76	F	IV	10.5	None
P47	62	M	II (L)	5.5	None	P83	63	F	IV	12	Intraperitoneal
P48	37	M	II (R)	5	None	P84	48	F	IV (L + R)	6.5 + 5 **	Intraperitoneal
P49	66	F	II (L)	5	None	P85	40	F	IV	10	None
P50	44	F	II (L)	5.5	None	P86	62	F	IV	11	None
P51	78	F	II (R)	8	None	P87	52	M	IV	11	None
P52	55	F	II (R)	4.5	None	P88	74	M	IV	14	Extraperitoneal
P53	42	M	II (L)	6	None	P89	60	F	IV (L + R)	8 + 7 **	Extraperitoneal
P54	64	M	II(R)	5	None	P90	45	M	IV	12	None
P55	54	F	II(L)	6.5	None	P91	50	F	IV (L + R)	6 + 7 **	Intraperitoneal
P56	43	F	II(R)	5	None	P92	84	F	IV	14	Intraperitoneal
P57	69	M	II(R)	6	None	P93	72	M	IV	14	Intraperitoneal
P58	94	F	II(L)	6.5	None	P94	37	M	IV	10	None
P59	63	M	II(L)	5.5	None						

* HB staging was carried out according to the International Society of Pediatric Oncology (SIOP) recommending PRE-Treatment EXTension (PRETEXT) system based on the imaging findings [3,31]; ** patient has two HB tumor lesions without any direct connection between them under gross or radiographic observation.

### 3.3. SELDI-TOF-MS Analysis

The serum samples were thawed in an ice bath and centrifuged at 10,000 rpm for 2 min at 4 °C. The supernatant was collected and 5 μL was mixed with 10 μL of U9 buffer (9 M urea, 2% CHAPS, and 1% DTT) and shaken at 600 rpm for 30 min at 4 °C. The serum samples were next diluted to 200 μL with NaAC (100 mM, pH 4) and shaken at 600 rpm for 30 min at 4 °C.

Prior to incubating with the serum samples, the WCX2 Protein Chip was pretreated with 200 μL of NaAC (100 mM, pH 4) and shaken at 600 rpm for 2 min at room temperature. After the protein chip was washed once more and shaken at 600 rpm for 2 min at room temperature, each well of the chip was loaded with 100 μL of pretreated serum sample and incubated at 600 rpm for 60 min at 4 °C. Each well of the protein chip was washed four times with 200 μL of NaAC (100 mM, pH 4) and shaken at 600 rpm for 5 min at room temperature. Each well was next washed with 200 μL of deionized water twice and spin-dried after which 1 μL of 50% saturated Sinapinic acid was added. The chip was then dried for subsequent analysis.

The following parameters were used for the SELDI-TOF-MS analysis: Maximum molecular weight, 30,000 Da; the best state, 2000 to 20,000 Da; laser intensity, 190; and sensitivity, 7. After the sample-mounted protein chip was analyzed by SELDI-TOF-MS, correction of the original data was carried out using Protein Chip Biomarker Software version 3.1 (Ciphergen Biosystems, Fremont, CA, USA). The data was analyzed using the Zhejiang University Protein Chip Data Analysis System (ZUCIPDAS). Various *m*/*z* values and corresponding protein peak values of the samples were obtained. Proteins with *m*/*z* difference less than 0.3% were considered to be the same type. The raw mass spectrometry data were treated using noise filtering and cluster analysis, and the *m*/*z* peaks obtained after initial screening were detected using the Wilcoxon rank-sum test. *p* < 0.01 was considered to be statistically significant. The mass spectrometry data from the different groups were compared using the independent samples *t*-test at a significance level of α = 0.01. A combined model with the highest Youden index was screened using SVM.

### 3.4. HPLC and MALDI-TOF-MS

Based on the SELDI-TOF-MS screening, serum samples with a relatively high expression level of target protein were selected for the isolation and purification. Briefly, 100 μL of serum sample was diluted with 300 μL of deionized water and 600 μL of Acetonitrile (ACN). After shaking, the serum sample was placed at 4 °C for precipitation of the serum proteins. After 30 min, the serum sample was centrifuged at 10,000 rpm for 30 min at 4 °C. The supernatant was concentrated under vacuum to a volume of less than 50 μL, mixed with 450 μL of 99.9% H_2_O/0.1% trifluoroacetic acid (TFA), and desalted using a self-packed C18 solid phase extraction column for HPLC. Separation of the pretreated serum samples was carried out by HPLC on a reverse-phase Phenomenex Onyx Monolithic C18 column. Mobile phase A (H_2_O/0.1% TFA) and mobile phase B (ACN/0.09% TFA) were applied for gradient elution as follows: 100% mobile phase A (15 min), 20%–40% gradient mobile phase B (15 min), 40%–70% gradient mobile phase B (50 min), and 100% mobile phase B (10 min). The flow rate was 0.5 mL/min, and the wavelengths were 214, 254 and 280 nm. The components of each peak were collected and concentrated under vacuum to a volume of less than 20 μL and analyzed by MALDI-TOF-MS.

### 3.5. Identification of the Target Protein

The purified target protein in a volume of 20 µL was combined with 60 μL of 8 M urea for a final concentration of 6 M urea and shaken at room temperature for 20 min. After addition of 0.8 μL of 1 M DTT, the sample was incubated at room temperature for 1 h, combined with 3.2 μL of 1 M iodoacetamide (IAM) in the dark for 45 min, followed by 3.2 μL of 1 M dl-dithiothreitol (DTT) at room temperature for 30 min, 400 μL of 50 mM NH_4_HCO_3_ and 0.08 μg trypsin for an overnight digestion at 37 °C. After 0.2% TFA was added to reduce the pH of the sample to <6.0 to terminate the enzymatic reaction, the sample was centrifuged at 12,000 rpm for 10 min. After the supernatant was concentrated under vacuum to a volume of less than 10 μL, it was added to a C18 gradient elution column for protein sample detection by 2D-LC-LTQ-MS. The peptide mass-to-charge ratio was analyzed using the SEQUEST program, and the target protein was identified using the Bioworks database.

### 3.6. ELISA

Expression levels of Apo A–I were detected in the serum of the normal group and the HB group. Lyophilized Apo A–I standards of various concentrations (0, 62.5, 125, 250, 500, 1000, 2000 and 4000 pg/mL) were added to a 96-well plate precoated with anti-human Apo A–I antibody (100 μL per well). All samples were analyzed in triplicate, and 10 serum samples were selected from each group. After a 100-fold dilution, 100 μL of the sample was added to each well for 90 min at 37 °C. After washing with PBS, the ABC working solution was added, and the ELISA plate was incubated for 60 min at 37 °C; then, the wells were washed, and 90 μL of TMB color development solution was added to each well. After incubation for 30 min at 37 °C, 100 μL of TMB substrate solution was added to each well to terminate the color development. The absorbance of each well was detected at 450 nm using Thermo Scientific Varioskan Flash. A standard curve was drawn according to the absorbance of the standard to calculate the actual concentrations of the samples.

## 4. Conclusions

In conclusion, Apo A–I was identified to be a specific serum protein marker of HB in the present study, and this marker may be an indicator of predicting HB stage. Further studies will include a larger number of samples to confirm its specificity and sensitivity and compare it with other diagnostic methods currently used in clinical practice. In addition, the relationship between Apo A–I and HB will be further explored to determine the effect of Apo A–I expression on the pathogenesis of HB.
